# Tramadol toxicity-induced rhabdomyolysis

**DOI:** 10.4103/0974-2700.70766

**Published:** 2010

**Authors:** Fahmi Yousef Khan, Hind Yousef, Mehdi Errayes

**Affiliations:** Department of Medicine, Hamad General Hospital, Doha, Qatar

Sir,

Rhabdomyolysis results from skeletal muscle injury with release of muscle cell contents into the plasma. It can occur from extensive muscle damage as, e.g., from a crushing injury or an electrical shock. Drugs or toxins such as cocaine, heroin, ethanol, amphetamines and caffeine may cause this disorder.[1,2] Tramadol is a rare cause of rhabdomyolysis; only few cases have been reported in the literature. In this report, we present a case of rhabdomyolysis in a 27-year-old man who ingested 1,000 mg of tramadol.

A 27-year-old Bahraini man, known to have depression and panic disorder, presented to the Emergency Department at Hamad General Hospital with lower back ache radiating to the right leg, associated with weakness. Two days prior to admission, the patient ingested 1,000 mg tramadol (10 tablets) during his flight from Bahrain to Doha to control his panic attacks in the air plane, after which he fell asleep at home for 36 h, lying on his right side. When he eventually woke up, he could not walk and he had a severe backache radiating to the right leg associated with numbness and weakness. He has no history of diabetes mellitus, hypertension or renal disease. He was not on regular medications.

On examination, he was obese (body mass index, 60), fully conscious and oriented, not dyspneic or pale, temperature 37°C, blood pressure 140/72 mmHg, pulse rate 110/min and respiratory rate 20/min. Neurological examination revealed right lower limb paresis (power 4/5) with right proximal muscle tenderness on palpation. Tone, reflexes, sensation and back and upper limb examination were all normal. The remaining examinations were unremarkable.

Laboratory investigations on admission revealed creatinine kinase (CK) of 19,926 U/L, aspartate aminotransferase 680 U/L, alanine aminotransferase 229 U/L, blood urea nitrogen 10.1 mmol/L, serum creatinine 375 μmol/L and serum myoglobin of 3,508 ng/ml. X-ray of the lumbar spine and ultrasound of the kidneys and pelvis were within normal limits.

The patient was diagnosed as tramadol toxicity-induced rhabdomyolysis and acute renal failure (ARF). He received vigorous intravenous fluid hydration. On the following days, he improved, the right lower limb pain and weakness resolved. On discharge, 4 days after hospitalization, the laboratory results including CK and serum creatinine were 1,444 U/L and 146 μmol/L respectively, with normal central nervous system (CNS) examination.

Tramadol is a centrally acting analgesic used for the treatment of moderate to severe pains. It has a weak affinity for the μ-opioid receptor and inhibits the re-uptake of norepinephrine and serotonin in the spine. The drug is rapidly absorbed orally and has a distribution volume of 3 L/kg. Following a 100 mg oral dose, a peak concentration of approximately 0.3 mg/L is detected 2 h post-dose. After a single bolus infusion of 100 mg tramadol, concentrations in plasma can be immediately detected. Elimination is slow and is characterized by an elimination half-life of 5–6 h.[3]

Thirty percent of the drug is excreted through the kidneys in an unchanged manner, while the remaining is metabolized by N- and O-demethylation, followed by conjugation with glucuronic acid and sulfate.[4]

Standard therapeutic doses are 50 mg orally, 50–100 mg by injection and 100 mg rectally. The total daily dose should not exceed 400 mg. Therapeutic blood levels in adults range from 0.1 to 0.3 mg/L, toxic level was between 1 and 2 mg/L and lethal concentration was usually considered to be higher than 2 mg/L, which suggests that therapeutic, toxic and lethal levels of tramadol were relatively close.[3] In our patient, although we did not check for tramadol blood level because of lack of facilities, the ingested dose (1,000 mg) was higher than the recommended dose (400 mg).

Tramadol overdose is associated with CNS and respiratory depression, coma, nausea and vomiting, tachycardia, agitation and seizures.[5] Although rare, tramadol toxicity is a recognized cause of rhabdomyolysis; it can induce rhabdomyolysis through a combination of mechanisms, including prolonged immobilization (due to CNS depression), and neuromuscular excitability (as serotonin syndrome). This patient had a history of prolonged immobilization after ingestion of 1,000 g of tramadol, but seizures cannot be excluded.

The clinical presentation of rhabdomyolysis is extremely variable due to the large range of causes. The classic manifestation of rhabdomyolysis includes acute myalgia and pigmenturia due to myoglobinuria in association with elevated serum muscle enzymes (CK in particular).[2] This patient presented with lower back ache radiating to the right leg, associated with weakness. He had no history of passing dark urine. Levels of CK in excess of five-times the normal are accepted as evidence of significant muscle breakdown and are generally considered to be consistent with a diagnosis of rhabdomyolysis.[1] Testing for serum or urine myoglobin is problematic and not always consistent. In this patient, serum CK and myoglobin were high (19,926 U/L and 3,508 ng/ml, respectively).

The complications of rhabdomyolysis include compartment syndrome, hyperkalaemia, hypocalcaemia, elevated liver enzymes, cardiac arrhythmias, ARF and disseminated intravascular coagulation. Therapy of rhabdomyolysis includes prompt and aggressive fluid resuscitation, elimination of causative agents and management and prevention of any complications that may arise. Alkalinization of the urine with sodium bicarbonate and diuresis with mannitol is unnecessary in patients with rhabdomyolysis and a good urinary response to fluid administration.[2] In the majority of the cases, hemodialysis was required before renal failure resolved. In our patient, however, ARF was resolved without hemodialysis.

Rhabdomyolysis affecting our patient was attributed to tramadol toxicity because he had not received any medications except tramadol before he fell asleep at home.

In conclusion, rhabdomyolysis is a rare but serious complication of tramadol toxicity that requires a high index of suspicion as the clinical manifestations are non-specific; therefore, physicians should be aware of this complication because any delay in the diagnosis and treatment may lead to renal failure and other life-threatening rhabdomyolysis-related complications.
